# Talazoparib, a Poly(ADP-ribose) Polymerase Inhibitor, for Metastatic Castration-resistant Prostate Cancer and DNA Damage Response Alterations: TALAPRO-1 Safety Analyses

**DOI:** 10.1093/oncolo/oyac172

**Published:** 2022-09-19

**Authors:** Niven Mehra, Karim Fizazi, Johann S de Bono, Philippe Barthélémy, Tanya Dorff, Adam Stirling, Jean-Pascal Machiels, Davide Bimbatti, Deepak Kilari, Herlinde Dumez, Consuelo Buttigliero, Inge M van Oort, Elena Castro, Hsiang-Chun Chen, Nicola Di Santo, Liza DeAnnuntis, Cynthia G Healy, Giorgio V Scagliotti

**Affiliations:** Department of Medical Oncology, Radboud University Medical Center, Nijmegen, The Netherlands; Institut Gustave Roussy, University of Paris-Saclay, Villejuif, France; The Institute of Cancer Research and The Royal Marsden Hospital, London, UK; Medical Oncology, Institut de Cancérologie Strasbourg Europe, Strasbourg, France; Medical Oncology & Therapeutics, City of Hope Comprehensive Cancer Center, Duarte, CA, USA; ICON Cancer Centre, Queensland, Australia; Medical Oncology, Cliniques Universitaires Saint-Luc, Brussels, Belgium; Medical Oncology, Université catholique de Louvain (UCLouvain), Belgium; Medical Oncology 1 Unit, Department of Oncology, Istituto Oncologico Veneto IOV IRCCS, Padova, Italy; Division of Hematology and Oncology, Medical College of Wisconsin, Milwaukee, WI, USA; Department of General Medical Oncology, University Hospitals Leuven, Leuven Cancer Institute, and Laboratory of Experimental Oncology, Department of Oncology, KU Leuven, Leuven, Belgium; Department of Oncology, University of Turin, San Luigi Gonzaga Hospital, Orbassano, Turin, Italy; Department of Urology, Radboud University Medical Center, Nijmegen, The Netherlands; Hospital Universitario Virgen de la Victoria, Instituto de Investigación Biomédica de Málaga (IBIMA), Málaga, Spain; Biostatistics, Pfizer Inc., La Jolla, CA, USA; Global Product Development, Pfizer Inc., Durham, NC, USA; Safety, Pfizer Inc., Collegeville, PA, USA; Oncology, Pfizer Inc., Collegeville, PA, USA; Department of Oncology, University of Turin, San Luigi Gonzaga Hospital, Orbassano, Turin, Italy

**Keywords:** *BRCA*, castration-resistant prostatic cancer, PARP inhibitor, talazoparib

## Abstract

**Background:**

The phase II TALAPRO-1 study (NCT03148795) demonstrated durable antitumor activity in men with heavily pretreated metastatic castration-resistant prostate cancer (mCRPC). Here, we detail the safety profile of talazoparib.

**Patients and Methods:**

Men received talazoparib 1 mg/day (moderate renal impairment 0.75 mg/day) orally until radiographic progression, unacceptable toxicity, investigator decision, consent withdrawal, or death. Adverse events (AEs) were evaluated: incidence, severity, timing, duration, potential overlap of selected AEs, dose modifications/discontinuations due to AEs, and new clinically significant changes in laboratory values and vital signs.

**Results:**

In the safety population (*N* = 127; median age 69.0 years), 95.3% (121/127) experienced all-cause treatment-emergent adverse events (TEAEs). Most common were anemia (48.8% [62/127]), nausea (33.1% [42/127]), decreased appetite (28.3% [36/127]), and asthenia (23.6% [30/127]). Nonhematologic TEAEs were generally grades 1 and 2. No grade 5 TEAEs or deaths were treatment-related. Hematologic TEAEs typically occurred during the first 4-5 months of treatment. The median duration of grade 3-4 anemia, neutropenia, and thrombocytopenia was limited to 7-12 days. No grade 4 events of anemia or neutropenia occurred. Neither *BRCA* status nor alteration origin significantly impacted the safety profile. The median (range) treatment duration was 6.1 (0.4-24.9) months; treatment duration did not impact the incidence of anemia. Only 3 of the 15 (11.8% [15/127]) permanent treatment discontinuations were due to hematologic TEAEs (thrombocytopenia 1.6% [2/127]; leukopenia 0.8% [1/127]).

**Conclusion:**

Common TEAEs associated with talazoparib could be managed through dose modifications/supportive care. Demonstrated efficacy and a manageable safety profile support continued evaluation of talazoparib in mCRPC.

**ClinicalTrials.gov identifier:**

NCT03148795

## Teaser

To aid clinicians in the management of adverse events and to inform clinical trials investigating the use of talazoparib in combination with other anticancer treatments, this article provides an in-depth analysis of the safety profile of talazoparib in men with metastatic castration-resistant prostate cancer.

**Implications for Practice**

In the TALAPRO-1 phase II trial, nonhematologic treatment-emergent adverse events were generally mild/moderate. Hematologic treatment-emergent adverse events were common but manageable through dose modifications and supportive care. The median duration of grade 3-4 anemia, neutropenia, and thrombocytopenia was limited to 7-12 days. No grade 4 events of anemia or neutropenia occurred. Permanent discontinuations due to adverse events occurred in slightly more than 1 in 10 patients (11.8%); 3 patients discontinued due to hematologic adverse events. Durable antitumor efficacy, coupled with the generally manageable safety profile, supports the further evaluation of talazoparib for men with metastatic castration-resistant prostate cancer.

## Introduction

Prostate cancer is the second most common cancer in men worldwide, with approximately 1.4 million new cases and 375 000 deaths in 2020.^1,2^ With improved detection and life expectancies, the global incidence of prostate cancer is expected to increase to 2.43 million cases in 2040.^1,3,4^ While androgen-deprivation therapy (ADT) induces remission for approximately 90% of patients with metastatic castration-sensitive prostate cancer (mCSPC), virtually all patients progress over time (median 24-36 months) and develop metastatic castration-resistant prostate cancer (mCRPC).^5-7^ The median survival time for patients with mCRPC is approximately 2-3 years.^6,8,9^ Despite declining mortality rates in most western countries, possibly due to early detection and treatment, the need for novel therapeutic strategies remains.^2,10,11^

Germline or somatic alterations in cellular DNA damage response (DDR) genes, involved directly or indirectly with homologous recombination repair (HRR), have been identified in 23%-27% of men with mCRPC.^12-15^ While these alterations are associated with worse clinical outcomes,^16-18^ they may sensitize patients to targeted therapies, such as poly(ADP-ribose) polymerase (PARP) inhibitors.^19^ Talazoparib inhibits PARP1 and PARP2, two key factors involved in DDR, and effectively traps PARP on single-stranded DNA breaks, causing an accumulation of double-stranded DNA breaks that cannot be effectively repaired in cancer cells with alterations in DDR-HRR genes such as *BRCA1* and *BRCA2*.^19-23^

Preclinical studies have also demonstrated that PARP2 plays a protective role against replicative stress in hematopoietic stem/progenitor cells, and depletion of PARP2 leads to anemia.^24,25^ PARP inhibitors are known to cause hematologic toxicities in patients with different tumor types.^26^ The phase III EMBRACA and phase II ABRAZO trials established the safety profile of talazoparib in female and male patients with advanced breast cancer.^27-30^ The most common hematologic toxicities were anemia, neutropenia, and thrombocytopenia.^28-31^

TALAPRO-1 is the first international phase II trial to evaluate the efficacy and tolerability of talazoparib monotherapy in heavily pretreated men with mCRPC and DDR-HRR alterations.^32,33^ The study demonstrated durable antitumor activity, with an objective response rate (ORR) of 29.8% (31/104; 95% CI, 21.2%-39.6%) observed for the primary cohort of patients with measurable disease. The highest efficacy was observed in men with *BRCA1/2* alterations (ORR = 45.9% [28/61]), although objective responses were observed in men with alterations in *PALB2* alone (ORR = 25.0% [1/4]) or *ATM* (ORR = 11.8 [2/17]). Adverse events observed in older men with mCRPC appeared similar to the established safety profile of talazoparib in younger women and men with breast cancer and included anemia, gastrointestinal events, and asthenia.^29,33^ Here, we provide an in-depth analysis of the specific safety profile of talazoparib in men with mCRPC that may aid clinicians in the management of AEs and inform clinical trials investigating the use of talazoparib in combination with other anticancer treatments.

## Patients and Methods

### Study Design and Participants

TALAPRO-1 (ClinicalTrials.gov identifier: NCT03148795) is an ongoing, multinational, open-label phase II trial. Detailed study information has been previously published^33^ but in summary, male patients (aged ≥18 years) with mCRPC were enrolled at 43 sites from within Australia, Brazil, Europe, South Korea, and the US. The safety population included patients with DDR-HRR core gene alterations that may sensitize to PARP inhibition (including but not limited to *ATM, ATR, BRCA1, BRCA2*, *CHEK2*, *FANCA*, *MLH1, MRE11A*, *NBN*, *PALB2*, *RAD51C*). Patients were previously treated with at least 1 taxane-based chemotherapy regimen for metastatic prostate cancer (castration-sensitive or -resistant) and progressed on at least 1 novel hormonal therapy (enzalutamide and/or abiraterone acetate/prednisone) given in the mCRPC setting.

All men received oral talazoparib (Pfizer Inc., New York, USA) 1 mg/day (0.75 mg/day for moderate renal impairment) and continued treatment until radiographic progression, unacceptable toxicity, investigator decision, withdrawal of consent, or death. Moderate renal impairment was defined as an estimated glomerular filtration rate of 30-59 mL/minute/1.73 m^2^ provided by the central laboratory. Appropriate dose modifications were allowed; dosage could be reduced in increments of 0.25 mg/day, and supportive care could be provided following grade 2, 3, or 4 events. For grade 3-4 hematologic AEs (defined as anemia, hemoglobin <80 g/L; neutropenia, absolute neutrophil count [ANC] <1000/µL; thrombocytopenia, platelets <50 000/µL), talazoparib was paused and the patient was monitored weekly. Upon resolution of the event (anemia, hemoglobin ≥90 g/L; neutropenia, ANC ≥1500/µL; and thrombocytopenia, platelets ≥50 000/µL), talazoparib was resumed at the next lowest dose level (as of protocol amendment 4 [November 15, 2018]). If the event persisted for >4 weeks without recovery, despite supportive care, talazoparib was permanently discontinued. Detailed protocol requirements following additional AEs have been previously published.^33^ Clinical laboratory tests and safety assessments were performed at screening and at each scheduled visit while taking talazoparib (every 2 weeks up to week 9, then every 4 weeks up to week 25, and thereafter every 12 weeks [hematology and serum chemistry every 8 weeks]).

TALAPRO-1 followed good clinical practice standards, the Declaration of Helsinki, and the International Conference on Harmonization. The Institutional Review Board or Ethics Committee at each study site approved the protocol. All patients provided signed informed consent.

### Outcomes

Outcomes and assessments have been previously published.^33^ The primary endpoint was confirmed ORR by blinded independent central review (BICR). Secondary and exploratory endpoints included time to objective response, duration of objective response, prostate-specific antigen (PSA) decline ≥50% from baseline, time to PSA progression, circulating tumor cell (CTC) conversion rate, radiographic progression-free survival per BICR and investigator assessment, overall survival, safety, and potential biomarkers of response. Patient-reported outcomes (time to deterioration in patient-reported pain, as assessed by the Brief Pain Inventory-Short Form [BPI-SF]; change from baseline in patient-reported pain as per the BPI-SF; and change from baseline in patient-reported outcome general health status, as assessed by the European Quality of Life 5-Dimension 5-Level Scale [EQ-5D-5L]) have been reported previously.^34^ The pharmacokinetic data will also be reported separately.

### Safety Assessments

Safety analyses comprised the incidence of serious and nonserious AEs (including incidence of new clinically significant changes in clinical laboratory values and vital signs), severity of AEs, timing and duration of AEs, and incidence of dose modifications and of permanent treatment discontinuation due to AEs. All AEs were evaluated using the National Cancer Institute-Common Terminology Criteria for Adverse Events, version 4.03, and coded using the Medical Dictionary for Regulatory Activities, version 23.0. Adverse events of special interest (AESI) included acute myeloid leukemia (AML), myelodysplastic syndrome (MDS), venous thrombotic events, pneumonitis, and second primary nonhematologic malignancies. The treatment-emergent period was defined as starting from the first dose of talazoparib and continuing until 28 days after the last dose, or before new systemic antineoplastic therapy or investigational drug, whichever occurred first. AEs were considered treatment-emergent AEs (TEAEs) if they occurred during the treatment-emergent period. For concurrent events, TEAEs were considered overlapping if the patient experienced both the AEs for at least 1 day. For analyses of anemia followed by fatigue, thrombocytopenia followed by bleeding event, and neutropenia followed by infections, the second AE (fatigue, bleeding, infection) had to start the same day or later after the first AE (anemia, thrombocytopenia, neutropenia), but the start date of the second AE was before the end date of the first AE.

### Statistical Analyses

The full statistical methodology has been previously reported.^33^ Most statistical analyses of the safety endpoints presented here are descriptive, although time-to-event endpoints are summarized using the Kaplan-Meier method and 95% CIs for medians are based on the Brookmeyer-Crowley method.

Data cutoff (at the primary completion date) was September 4, 2020. All safety analyses were performed on the safety population, which was defined as all men who received ≥1 dose of talazoparib, including a subset of men with nonmeasurable disease enrolled under an early version of the protocol and DDR-HRR gene alterations assessed using an expanded DDR-HRR gene panel (FoundationOne^®^ CDx; Foundation Medicine, Cambridge, MA) including genes likely to sensitize to PARP inhibition.

For endpoints analyzed by DDR-HRR alteration, a hierarchical strategy, implemented post hoc based on the latest understanding of the likely relative importance of the alteration, was used. Men were separated into alteration groups in the following order: *BRCA1/2* ranked above *PALB2*, *PALB2* ranked above *ATM*, and *ATM* ranked above all other alterations. Association between hematologic AEs and germline DDR-HRR alteration was examined as a post hoc analysis. *P* values were based on Fisher’s exact test.

## Results

### Patients

The safety population comprised 127 men (median age 69.0 years) with at least one DDR-HRR alteration enrolled between July 4, 2017, and March 20, 2020, from an initial population that included 1425 screened men with mCRPC (1297 patients either did not have DDR-HRR alterations or failed to meet other eligibility criteria and one patient did not receive talazoparib) (Supplementary Fig. S1). Of the 127 men included in the safety population, 23 (18.1%) were enrolled under earlier versions of the protocol and did not have measurable disease, and may have had alterations in genes from the expanded screening panel. Baseline characteristics are summarized in Table 1, including the incidence of DDR-HRR alterations. In the safety population, single DDR-HRR alterations were detected in 113 (89%) men and 14 (11%) had alterations in more than one DDR-HRR gene. Of the 127 men included in the safety population, *BRCA2* alterations were the most common, detected in 48% (61) of men, followed by alterations in *ATM* (15.7% [20]), *CHEK2* (9.4% [12]), *PALB2* (5.5% [7]), and *BRCA1* and *MLH1* (each 4.7% [6]). Five (3.9%) men had alterations detected in *MSH2*; 4 (3.1%) in *NBN*; 3 (2.4%) each in *FANCA* and *RAD51C*; 2 (1.6%) each in *ATR, FANCD2*, *MUTYH*, and *RAD50*; and 1 (0.8%) each in *ERCC4*, *FANCE*, *MRE11A*, *MSH6*, *POLD1*, *RAD51B*, and *TP53BP1.*

**Table 1.** Baseline patient characteristics (safety and efficacy populations).^a^

	Safety population (*N* = 127)	Efficacy population					
		*BRCA1/2* (*N* = 61)^b^	*BRCA2* (*N* = 57)^b^	*PALB2* (*N* = 4)	*ATM* (*N* = 17)^c^	Other (*N* = 22)^d^	Total (*N* = 104)
Age, median (range), years	69.0 (46.0-84.0)	69.0 (46.0-83.0)	69.0 (46.0-83.0)	72.5 (51.0-79.0)	67.0 (53.0-81.0)	71.0 (57.0-82.0)	69.0 (46.0-83.0)
Race, *n* (%)							
White	110 (86.6)	53 (86.9)	50 (87.7)	3 (75.0)	16 (94.1)	19 (86.4)	91 (87.5)
Black	4 (3.1)	3 (4.9)	3 (5.3)	0	0	0	3 (2.9)
Asian	3 (2.4)	0	0	0	1 (5.9)	1 (4.5)	2 (1.9)
Not reported	10 (7.9)	5 (8.2)	4 (7.0)	1 (25.0)	0	2 (9.1)	8 (7.7)
Renal impairment, *n* (%)							
Normal/mild	105 (82.7)	50 (82.0)	46 (80.7)	4 (100.0)	15 (88.2)	16 (72.7)	85 (81.7)
Moderate	22 (17.3)	11 (18.0)	11 (19.3)	0	2 (11.8)	6 (27.3)	19 (18.3)
Baseline serum PSA (µg/L), median (IQR)	103.8 (24.0-303.1)	97.4 (19.4-299.5)	97·4 (20.8-296.0)	118.7 (56.3-244.5)	178.5 (56.1-308.5)	153.5 (33.8-305.0)	118.6 (26.0-304.1)
Baseline testosterone (ng/dL), median (IQR)	10.1 (10.1-19.9)	10.1 (10.1-15.8)	10.1 (10.1-15.0)	15.8 (10.0-33.4)	14.1 (10.1-22.2)	10.1 (10.1-20.7)	10.1 (10.1-17.7)
Baseline CTC count (cells/7.5 mL of blood), median (IQR)	5.0 (0.0-41.0)	5·0 (0.0-22.0)	5.0 (0.0-22.0)	13.0 (3.0-490.0)	23.0 (0.0-62.0)	3.5 (1.5-36.5)	5.0 (1.0-38.0)
Total Gleason score, *n* (%)							
Grade Group 1 (≤6)	9 (7.1)	4 (6.6)	4 (7.0)	0	2 (11.8)	3 (13.6)	9 (8.7)
Grade Group 2 (3 + 4) and 3 (4 + 3)	39 (30.7)	17 (27.9)	17 (29.8)	0	6 (35.3)	8 (36.4)	31 (29.8)
Grade Groups 4 (8) and 5 (9-10)	78 (61.4)	39 (63.9)	35 (61.4)	4 (100.0)	9 (52.9)	11 (50.0)	63 (60.6)
Not reported	1 (0.8)	1 (1.6)	1 (1.8)	0	0	0	1 (1.0)
Initial M stage at primary diagnosis, *n* (%)							
M0	50 (39.4)	26 (42.6)	24 (42.1)	0	6 (35.3)	7 (31.8)	39 (37.5)
M1	57 (44.9)	26 (42.6)	25 (43.9)	4 (100.0)	9 (52.9)	9 (40.9)	48 (46.2)
MX	16 (12.6)	7 (11.5)	6 (10.5)	0	1 (5.9)	5 (22.7)	13 (12.5)
Not reported	4 (3.1)	2 (3.3)	2 (3.5)	0	1 (5.9)	1 (4.5)	4 (3.8)
Disease site, *n* (%)							
Visceral	41 (32.3)	18 (29.5)	17 (29.8)	3 (75.0)	5 (29.4)	10 (45.5)	36 (34.6)
Non-visceral	86 (67.7)	43 (70.5)	40 (70.2)	1 (25.0)	12 (70.6)	12 (54.5)	68 (65.4)
ECOG performance status, *n* (%)							
0	52 (40.9)	24 (39.3)	22 (38.6)	0	8 (47.1)	9 (40.9)	41 (39.4)
1	63 (49.6)	31 (50.8)	29 (50.9)	4 (100.0)	8 (47.1)	10 (45.5)	53 (51.0)
2	12 (9.4)	6 (9.8)	6 (10.5)	0	1 (5.9)	3 (13.6)	10 (9.6)
Prior taxane use, *n* (%)							
Docetaxel only	65 (51.2)	35 (57.4)	32 (56.1)	1 (25.0)	9 (52.9)	9 (40.9)	54 (51.9)
Docetaxel and cabazitaxel	61 (48.0)	26 (42.6)	25 (43.9)	3 (75.0)	8 (47.1)	12 (54.5)	49 (47.1)
Not reported	1 (0.8)	0	0	0	0	1 (4.5)	1 (1.0)
Prior NHT, *n* (%)							
Abiraterone acetate only	45 (35.4)	28 (45.9)	27 (47.4)	2 (50.0)	3 (17.6)	4 (18.2)	37 (35.6)
Enzalutamide only	46 (36.2)	20 (32.8)	19 (33.3)	2 (50.0)	8 (47.1)	7 (31.8)	37 (35.6)
Abiraterone acetate and enzalutamide	34 (26.8)	12 (19.7)	10 (17.5)	0	6 (35.3)	10 (45.5)	28 (26.9)
Not reported	2 (1.6)	1 (1.6)	1 (1.8)	0	0	1 (4.5)	2 (1.9)

Reprinted from de Bono et al.^33^ Copyright (2021), with permission from Elsevier.

Patients were separated hierarchically by HRR gene alterations with *BRCA1/2* ascendant over *PALB2*, *PALB2* ascendant over *ATM*, and *ATM* ascendant over all other alterations.

*BRCA1/2* and *BRCA2*: included two patients with both *BRCA2* and *PALB2* alterations, one patient with both *BRCA2* and *ATM* alterations, one patient with both *BRCA2* and *CHEK2* alterations, and one patient with both *BRCA2* and *MLH1* alterations.

*ATM*: included one patient with both *ATM* and *FANCA* alterations, and one patient with both *ATM* and *RAD51C* alterations.

Other: *ATR*, *CHEK2*, *FANCA*, *MLH1*, *MRE11A*, *NBN*, and *RAD51C.*

Abbreviations: *BRCA1/2, breast cancer susceptibility gene 1 or 2*; CTC, circulating tumor cell; ECOG, Eastern Cooperative Oncology Group; HRR, homologous recombination repair; IQR, interquartile range; n, number; NHT, novel hormonal therapy; PSA, prostate-specific antigen.

Within the safety population, there were 37 (29.1%) men who received talazoparib for between ≥6 months and <12 months and 28 (22.0%) men who received talazoparib for ≥12 months. The median (range) treatment duration of talazoparib was 6.1 (0.4-24.9) months for all men and 8.3 (0.9-22.2) months for men with *BRCA1/2* alterations.

### Occurrence of Treatment-Emergent Adverse Events

**Figure 1.**
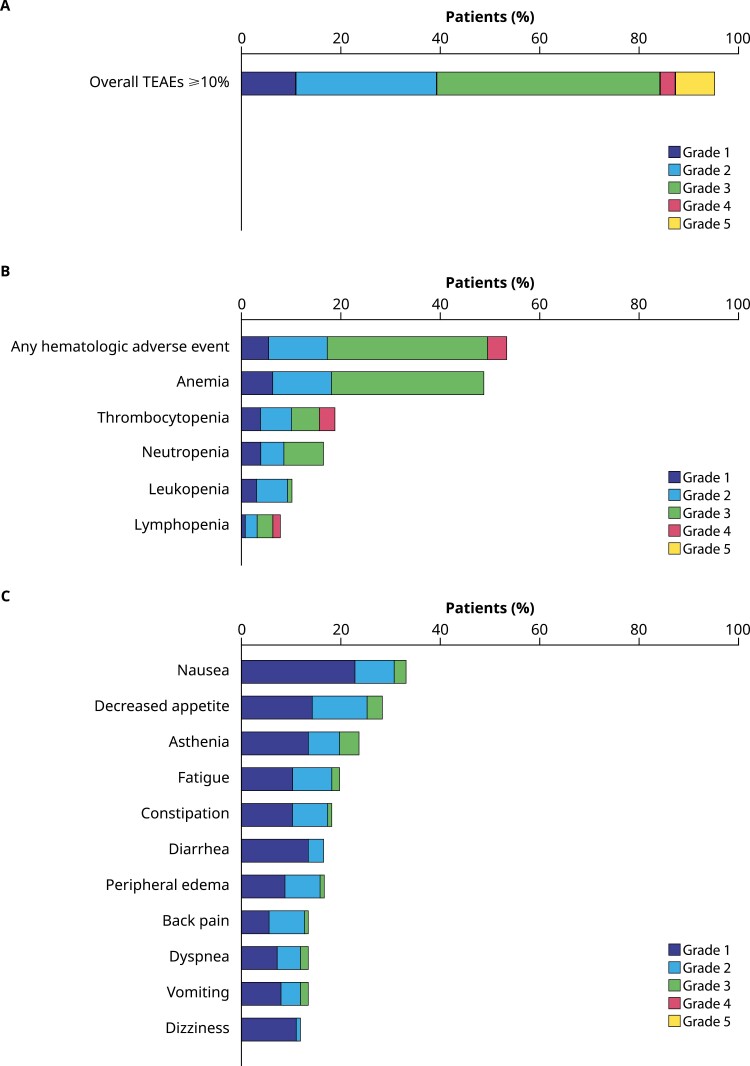
Any grade TEAEs **(A)** overall, **(B)** hematologic, and **(C**) nonhematologic^a^ (*N* = 127; safety population). Data were included up to 28 days after the last dose of talazoparib or before new systemic antineoplastic therapy, whichever occurred first. MedDRA v23.0 coding dictionary applied; NCI-CTCAE version 4.03. ^a^Includes TEAEs experienced by ≥10% of patients. Abbreviations: MedDRA, Medical Dictionary for Regulatory Activities; NCI-CTCAE, National Cancer Institute-Common Terminology Criteria for Adverse Events; TEAE, treatment-emergent adverse event.

In the safety population, 95.3% (121/127) of all men experienced all-cause TEAEs (Fig. 1A). The most common any grade hematologic AEs in the safety population included anemia (48.8% [62/127]), thrombocytopenia (18.9% [24/127]), and neutropenia (16.5% [21/127]) (Fig. 1B). As shown in Fig. 1C, the most commonly reported nonhematologic AEs in the safety population included nausea (33.1% [42/127]), decreased appetite (28.3% [36/127]), and asthenia (23.6% [30/127]). Most nonhematologic AEs were grades 1 and 2.

None of the grade 5 TEAEs (7.9% [10/127]) were related to the study drug, and they included death due to disease progression (4.7% [6/127]), general physical health deterioration (0.8% [1/127]), pulmonary embolism (0.8% [1/127]), subdural hematoma (0.8% [1/127]), and cardio-respiratory arrest (0.8% [1/127]).

All-cause serious TEAEs were reported in 43 (33.9%) men (Supplementary Table S1). The most common (in >1 man) were pulmonary embolism (6.3% [8/127]), anemia (3.9% [5/127]), and pneumonia and urinary tract infection (both 2.4% [3/127]). Disease progression, reported as an AE, occurred in 3.1% [4/127] of men. There were 17 (13.4%) instances of any grade AESI, which included venous thrombotic events (9.4% [12/127]), nonhematologic second primary malignancies (2.4% [3/127]), and pneumonitis (1.6% [2/127]). No men had AML or MDS while on study or by the end of follow-up. There were no treatment-related deaths (Supplementary Table S2).

### Onset and Duration of AEs Associated with Talazoparib

**Figure 2.**
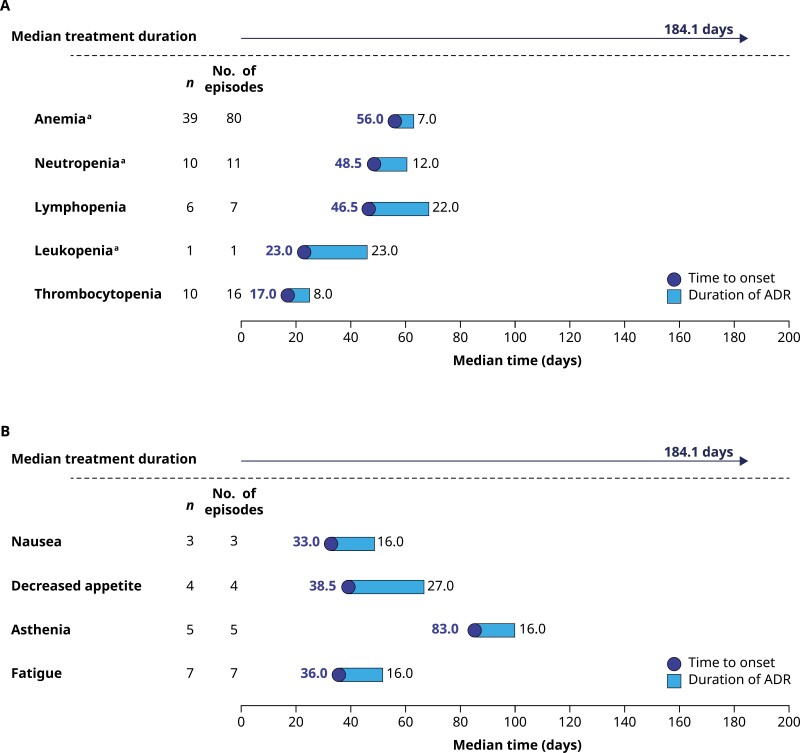
Median time from first dose of talazoparib to onset and duration of first grade ≥3 TEAEs: **(A)** hematologic adverse drug reactions and **(B)** nonhematologic adverse drug reactions (*N* = 127; safety population). MedDRA v23.0 coding dictionary applied; grades are evaluated based on NCI-CTCAE (version 4.03). Maximum grade of cluster/preferred term is considered for presenting descriptive statistics under the maximum grade of cluster/preferred term section. For duration of ADR by grade, all episodes with grades within the range were used in the calculation of descriptive statistics. ^a^No grade 4 TEAEs for anemia, neutropenia, or leukopenia; 2 men (1.6%) had grade 4 lymphopenia (median time to onset 75 days and duration 14 days) and 4 men (3.1%) had grade 4 thrombocytopenia (median time to onset 14.5 days and duration 11 days). Abbreviations: ADR, adverse drug reaction; MedDRA, Medical Dictionary for Regulatory Activities; NCI-CTCAE, National Cancer Institute-Common Terminology Criteria for Adverse Events; TEAE, treatment-emergent adverse event.

The median time (range) from the first dose of talazoparib to the onset of the first episode of grade 3 or higher anemia, neutropenia, and thrombocytopenia was 56.0 (13.0-198.0), 48.5 (15.0-263.0), and 17.0 (13.0-116.0) days, respectively. The median (range) duration of grade 3 anemia, grade 3 neutropenia, grade 3 thrombocytopenia, and grade 4 thrombocytopenia was 7 (1-38), 12 (7-18), 8 (5-28), and 11 (8-17) days, respectively (Fig. 2A). No grade 4 events of anemia or neutropenia occurred. The median time (range) from the first dose of talazoparib to the onset of the first episode of grade 3 nausea, decreased appetite, asthenia, and fatigue was 33.0 (13.0-49.0), 38.5 (30.0-50.0), 83.0 (13.0-737.0), and 36.0 (2.0-737.0) days, respectively (Fig. 2B). Definitions of hematologic AE grades are provided in Supplementary Table S3.^35^

**Figure 3.**
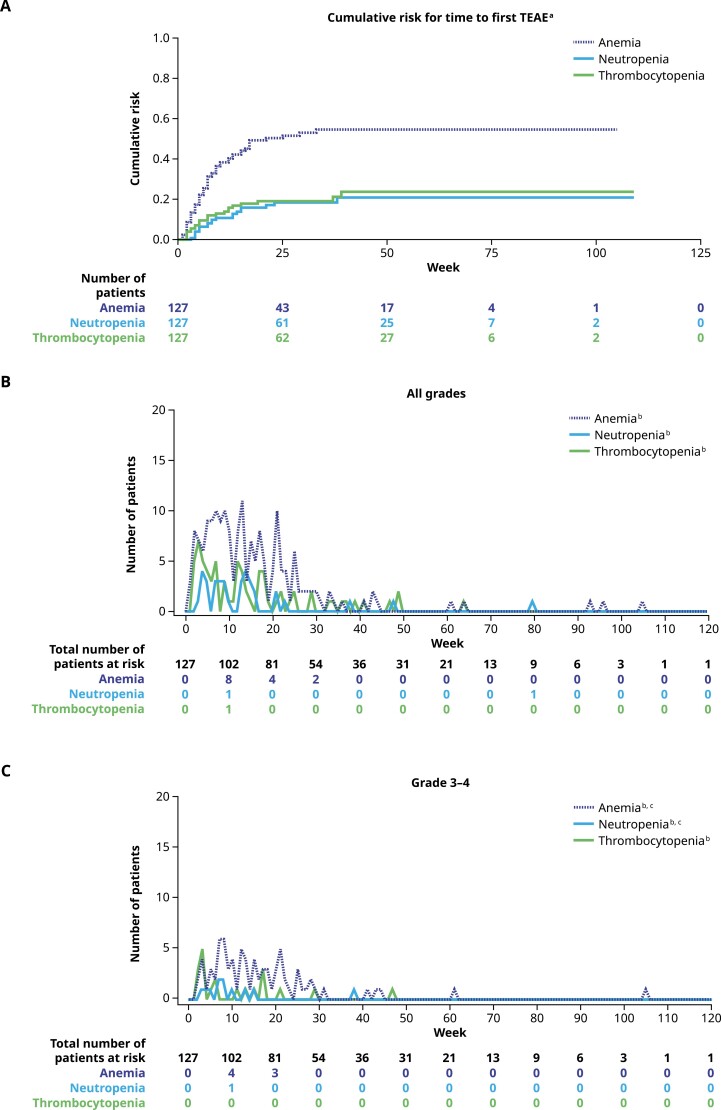
**(A)** Cumulative risk by week for time to first TEAE for anemia, neutropenia, and thrombocytopenia (cluster terms), **(B)** all grade TEAEs by week, and **(C)** grades 3-4 TEAEs by week (*N* = 127; safety population). Within each week, men with new reports of TEAEs within the cluster term were counted. Cluster terms were defined as ANEMIA: anemia, hematocrit decreased, hemoglobin decreased, red blood cell count decreased; NEUTROPENIA: neutropenia, neutrophil count decreased; THROMBOCYTOPENIA: thrombocytopenia, platelet count decreased. MedDRA v23.0 coding dictionary was applied. ^a^Time to first episode of a preferred term within the cluster term was evaluated. Men who did not experience an event were censored at the end of the TE period. ^b^Number of patients with TEAEs. ^c^No grade 4 events occurred. Abbreviations: MedDRA, Medical Dictionary for Regulatory Activities; TE, treatment-emergent; TEAE, treatment-emergent adverse event.

**Figure 4.**
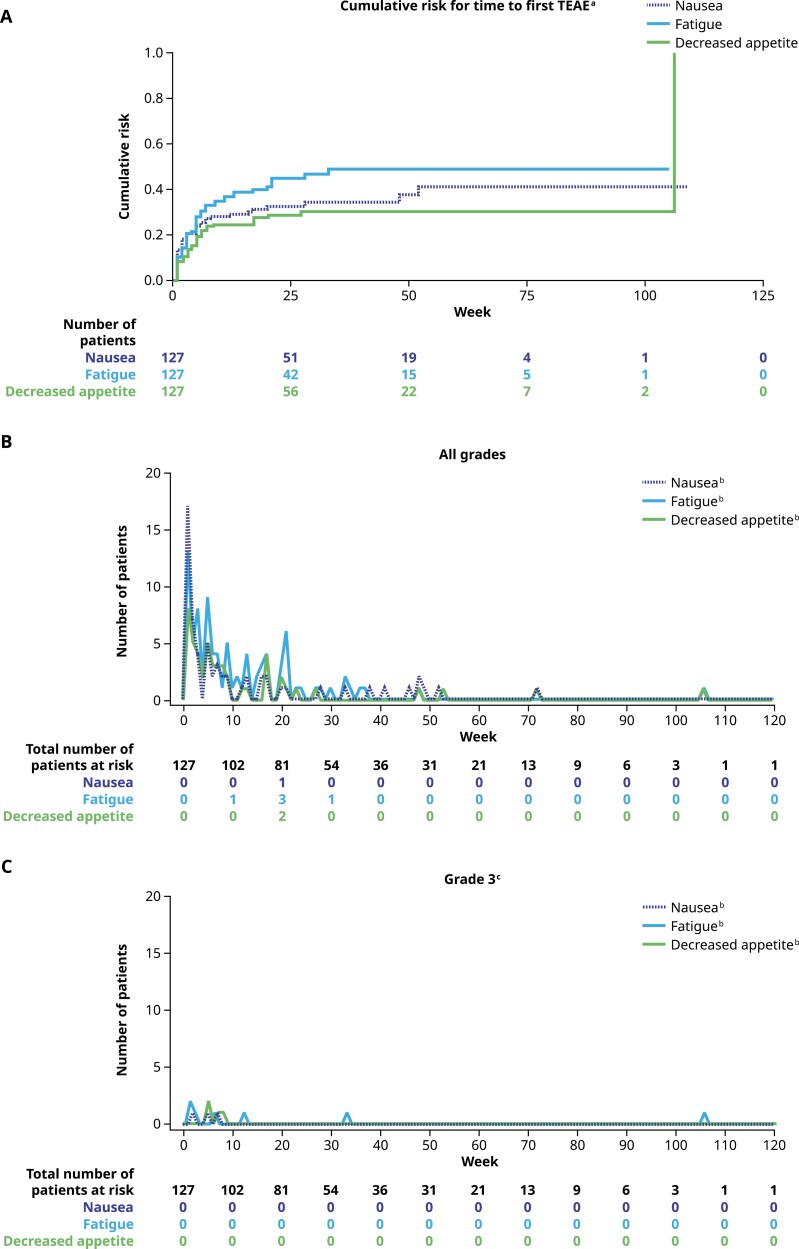
**(A)** Cumulative risk by week for time to first TEAE for nausea, fatigue (cluster term), and decreased appetite, **(B)** all grade TEAEs by week, and **(C)** grade 3 TEAEs by week (*N* = 127; safety population). MedDRA v23.0 coding dictionary was applied. Within each week, men with new reports of TEAEs within the cluster term were counted. Cluster term defined as FATIGUE: fatigue and asthenia. One patient had first occurrence of decreased appetite at 106 weeks after treatment start. ^a^Time to first episode of a preferred term within the cluster term was considered. Men who did not experience an event were censored at the end of the treatment-emergent period. ^b^Number of patients with TEAEs. ^c^There were no grade 4 or 5 TEAEs observed for nausea, fatigue, or decreased appetite. Abbreviations: MedDRA, Medical Dictionary for Regulatory Activities; TEAE, treatment-emergent adverse event.

The cumulative risk by week for the most common hematologic TEAEs is shown in Fig. 3A. As shown in Fig. 3B and 3C, hematologic TEAEs typically occurred during the first 4-5 months of treatment. The cumulative risk by week for the most common nonhematologic TEAEs is presented in Fig. 4A. A similar trend for the onset of the most common all grade nonhematologic TEAEs was also observed (all grades, Fig. 4B; grade 3, Fig. 4C).

TEAEs were managed by dose modifications and supportive care. In the safety population, 34.6% (44/127) of men received ≥1 concomitant blood transfusion product and 29.9% (38/127) received a packed red blood cell transfusion (Supplementary Table S4); of those who received a packed red blood cell transfusion, 88.9% had hemoglobin levels between 70.0-110.0 g/L prior to the first transfusion (Supplementary Fig. S2). Of the 48.8% (62/127) of men who experienced anemia, 72.6% (45/62) received ≥1 supportive treatment. Overlapping grade 3-4 hematologic TEAEs were infrequent in men receiving talazoparib. The most common overlapping grade 3-4 condition was fatigue following the occurrence of anemia (12.6% [16/127]; Supplementary Fig. S3).

### Impact of Clinical and Tumor Characteristics on Hematologic TEAEs

Neither *BRCA* status nor DDR-HRR gene alteration origin significantly impacted the development of grade 3-4 anemia. The baseline characteristics, prior treatments, germline or somatic DDR-HRR alteration status, and duration of treatment within the overall safety population, patients with grade 3-4 anemia, and patients with grade 3-4 neutropenia are shown in Supplementary Fig. S4. The duration of treatment was similar in the overall population versus men with grade 3-4 anemia. There were 51.2% (65/127) of men in the overall population who received treatment for 6 months or longer versus 56.4% (22/39) of men with grade 3-4 anemia. Among men with grade 3-4 anemia, 56.4% (22/39) had *BRCA1/2* alterations and 72.7% (16/22) of these men had a duration of treatment 6 months or longer versus 56.4% (22/39) of men in the overall population with grade 3-4 anemia.

The impact of various baseline characteristics on the development of treatment-emergent grade 3-4 anemia or neutropenia was examined. Men with low baseline hemoglobin (<100 g/L) were more likely to experience treatment-emergent grade 3-4 anemia (75% [15/20]) than those with baseline hemoglobin within normal limits (22.4% [24/107]; *P* < .0001). Men with 4 or more prior anticancer regimens experienced a higher incidence of treatment-emergent grade 3-4 anemia (35.6% [26/73]) than men with 1-3 prior anticancer regimens (24.5% [13/53]), although the difference was not significant (*p* =.24). Duration of treatment was not significantly associated with the incidence of treatment-emergent grade 3-4 anemia: 27.4% (17/62) who received treatment for less than 6 months developed treatment-emergent grade 3-4 anemia versus 33.8% (22/65) who received treatment for no less than 6 months. The incidence of any grade anemia was similar between men with normal renal function (48.5% [32/66]) and mild renal impairment (43.6% [17/39]), and the incidence was higher in men with moderate renal impairment (59.1% [13/22]).

For men with fewer than 10 baseline bone metastases, the associated risk of developing any grade of anemia was 44.2% ([19/43]; the risk of these patients presenting with grade ≥3 anemia was 23.3% [10/43]). For men with at least 10 bone metastases at baseline, the incidence was 62.5% ([35/56] for developing any grade of anemia and 42.9% [24/56]) for developing grade ≥3 anemia (Supplementary Fig. S5).

### Dose Modifications and Discontinuations

Dose interruptions or reductions due to TEAEs occurred in 47 (37.0%) and 33 (26.0%) men, respectively. The most common TEAEs leading to dose interruptions (>2 men) were anemia (18.9% [24/127]), thrombocytopenia (11.0% [14/127]), neutropenia (7.9% [10/127]), and decreased appetite (4.7% [6/127]). The most common TEAEs leading to dose reductions (>1 patient) were anemia (22.0% [28/127]), thrombocytopenia (4.7% [6/127]), neutropenia (3.1% [4/127]), and lymphopenia ([2.4% [3/127]) (Supplementary Table S5). Of the 23 (18.1%) men who experienced grade 3-4 anemia leading to a dose reduction, 10 (43.5%) experienced a recurrence of grade 3-4 anemia (Supplementary Table S6). Of the 32 (25.2%) men who experienced grade 3-4 anemia leading to a transfusion, 15 (46.9%) experienced a recurrence of grade 3-4 anemia (Supplementary Table S6).

Permanent treatment discontinuations due to all-causality TEAEs occurred in 15 men (11.8%); median (range) duration of treatment was 1.97 (0.43-7.92) months. The majority of permanent discontinuations were due to nonhematologic AEs, including back pain (1.6% [2 men]), and cancer pain, cardio-respiratory arrest, and disease progression (0.8% [1 man] each). Two (1.6%) men discontinued due to decreased platelet count and 1 (0.8%) man discontinued due to a decreased white blood cell count (Supplementary Table S5).

## Discussion

The TALAPRO-1 trial demonstrated talazoparib to be effective in the treatment of mCRPC, particularly in men with *BRCA1/2* alterations.^33^ The TALAPRO-1 patient population (older, heavily pretreated men with mCRPC) can be difficult to treat.^36-38^ Although the populations are distinct (patients with breast cancer were on average younger than patients with prostate cancer), the TEAEs were consistent with the established safety profile for talazoparib in female and male patients with germline *BRCA* (g*BRCA*)-altered advanced breast cancer.^27-30,39^ Here, we detail the safety profile of talazoparib in heavily pretreated men with mCRPC and somatic or germline DDR-HRR alterations to inform dosing in future clinical trials and provide clinicians with a thorough understanding of the incidence and management of TEAEs associated with talazoparib treatment.

The phase III EMBRACA and phase II ABRAZO trials established the safety profile of talazoparib in female and male patients with advanced breast cancer.^27-30^ In the EMBRACA trial, which led to the approval of talazoparib for the treatment of adult female and male patients with g*BRCA1/2*-altered human epidermal growth factor receptor 2-negative metastatic or locally advanced breast cancer, the most common hematologic toxicities were anemia, neutropenia, and thrombocytopenia.^28,29,31^ The most common nonhematologic AEs were fatigue, nausea, headache, alopecia, and vomiting.^29^ In the ABRAZO trial, patients with advanced breast cancer and g*BRCA1/2* alterations, who had previously received either platinum or platinum-free cytotoxic regimens, demonstrated a similar safety profile. The most common AEs reported were anemia, fatigue, and nausea.^30^ In the EMBRACA and ABRAZO trials, the median (range) age of patients was 45 (27-84) years and 50 (31-75) years, respectively,^29,30^ versus 69.0 (interquartile range [IQR] 63.0-74.0) years in the TALAPRO-1 trial.^33^ All men in the TALAPRO-1 trial had received 1 or 2 prior chemotherapy regimens, including 1 or more taxane-based regimen in the metastatic setting. In contrast, in the EMBRACA trial, 76% of patients had received ≤1 prior cytotoxic regimen for advanced breast cancer.^29^ The ABRAZO population was more heavily pretreated: a median (range) of 3 (1-10) prior cytotoxic therapies for advanced disease.^30^

PARP inhibitors are known to cause hematologic toxicities, and anemia (48.8%) was the most common any grade hematologic toxicity in the TALAPRO-1 population. This finding is consistent with the incidence of anemia reported in patients with *gBRCA*-altered advanced breast cancer treated with talazoparib in the EMBRACA trial (any grade, 52.8%) and ABRAZO trial (any grade, 52%).^28,30^ In TALAPRO-1, hematologic TEAEs were generally well-managed through dose modifications and supportive care. Of the 62 men who experienced anemia, 72.6% (45/62) received ≥1 supportive treatment and anemia was the most common TEAE leading to dose interruptions or reductions. With supportive care, the median duration of the most common grade 3 hematologic events was limited to 7-12 days. In the EMBRACA and ABRAZO trials, anemia was also the most common cause leading to a dose reduction or temporary interruption.^29,30^ However, there were no permanent discontinuations due to anemia in the TALAPRO-1 study. In the PROfound trial, 21.5% (55/256) of men (median [range] age 69 [47-91] years) treated with olaparib for mCRPC experienced grade ≥3 anemia.^40^ In the GALAHAD trial, 33% (95/289) of men (median [IQR] age 69.0 [64.0-74.0] years) with mCRPC treated with niraparib experienced grade ≥3 anemia.^41^

No significant differences in the safety profile based on *BRCA* status or germline versus somatic alteration origin were observed. Ledermann et al also reported no significant difference in the proportion (or incidence) of patients with anemia between the overall patient population (any grade, 21%; grade ≥3, 5%) and those with *BRCA1/2* alterations (any grade, 26%; grade ≥3, 5%) in patients with ovarian cancer treated with olaparib.^42^ However, in the TRITON2 trial, which examined rucaparib in men with mCRPC, anemia was found to be more common in men with *BRCA1/2* alterations (any grade, 43.5%; grade ≥3, 25.2%) than men with deleterious non-*BRCA* DDR alterations (any grade, 30.8% [24/78]; grade ≥3, 15.4% [12/78]).^43,44^ In the TRITON2 trial, the median age (range) of patients with *BRCA* alterations was 72.0 (50-88), median number (range) of prior therapies for CRPC was 2 (1-8), and the proportion of patients who had ≥10 bone metastases was 47.0% (54/115).^44^ The median age (IQR) for patients with *ATM*, *CDK12*, *CHEK2*, and other alterations in the TRITON2 trial was 73.0 (68.0-77.0), 64.0 (56.0-72.0), 71.5 (64.5-75.0), and 66.5 (61.0-72.0) years, respectively, and the median number of prior CRPC treatments for non-*BRCA* patients ranged between 2 and 3 (IQR 2-4).^43^

In TALAPRO-1, the most common any grade nonhematologic TEAEs were nausea (33.1% [42/127]), decreased appetite (28.3% [36/127]), asthenia (23.6% [30/127]), and fatigue (19.7% [25/127]); most events were grade 1 or 2 in severity. Serious AEs due to nonhematologic TEAEs were less common. In the EMBRACA trial, the most common nonhematologic TEAEs associated with talazoparib were fatigue (62.2% [178/286]) and nausea (48.5% [139/286]) in women with g*BRCA* alterations and advanced breast cancer. Any grade alopecia and vomiting occurred in 25.2% (72/286) and 24.8% (71/286) of patients.^28^ However, in TALAPRO-1, the incidence of alopecia was lower than the ≥10% cutoff (4.7% [6/127]) and 13.4% (17/127) of men experienced vomiting.

Higher talazoparib exposure is associated with a higher incidence of hematologic AEs and dose interruptions, and reductions are an effective management strategy.^27^ In TALAPRO-1, dose reductions due to an AE were common (26.0%); while permanent discontinuations due to an AE occurred in 11.8% of patients, only 2.4% of discontinuations were due to hematologic AEs. This finding is consistent with the trend observed in the EMBRACA trial (reductions due to an AE, 52.1%; discontinuations due to an AE, 5.9%; discontinuations due to a hematologic AE, 1.4%).^28,29^

A thorough understanding of the safety profile for treatment with talazoparib monotherapy and management of AEs in men with metastatic prostate cancer is essential in making treatment decisions and in future studies where there may be drug-drug interactions to consider. There are currently two ongoing phase III trials that compare talazoparib plus enzalutamide with enzalutamide monotherapy in men with metastatic prostate cancer: TALAPRO-2 and TALAPRO-3. TALAPRO-2 (NCT03395197) is studying men with mCRPC, with and without DDR-HRR alterations in a first-line setting. Following the conclusion of Part 1 of the TALAPRO-2 trial, the recommended dose of talazoparib was 0.5 mg/day (0.35 mg/day if moderate renal impairment) due to drug-drug interactions when combined with enzalutamide (160 mg/day).^45^ TALAPRO-3 (NCT04821622) is enrolling men with mCSPC and DDR-HRR alterations receiving either 0.5 mg talazoparib plus 160 mg enzalutamide, or enzalutamide monotherapy.

## Conclusion

In the ongoing TALAPRO-1 phase II study, no new safety signals were observed with talazoparib monotherapy in men with heavily pretreated mCRPC with DDR-HRR alterations. The most common any-grade TEAEs were anemia, nausea, decreased appetite, and asthenia. TEAEs were not associated with germline compared with somatic alterations. TEAEs were well managed through dose modifications and supportive care, with grade 3 hematologic AEs having a median duration of 7-12 days for the 3 most common hematologic AEs. The manageable safety profile and observed antitumor activity support further evaluation of talazoparib in advanced prostate cancer.

## Supplementary Material

oyac172_suppl_Supplementary_Figure_1

oyac172_suppl_Supplementary_Figure_2

oyac172_suppl_Supplementary_Figure_3

oyac172_suppl_Supplementary_Figure_4

oyac172_suppl_Supplementary_Figure_5

oyac172_suppl_Supplementary_Figures

oyac172_suppl_Supplemental_Tables_1_6_final

## Data Availability

Upon request and subject to review, Pfizer will provide the data that support the findings of this study. Subject to certain criteria, conditions, and exceptions, Pfizer may also provide access to the related individual de-identified participant data. See https://www.pfizer.com/science/clinical-trials/trial-data-and-results for more information.
