# Supplementary figures and images for: Talazoparib, a Poly(ADP-ribose) Polymerase Inhibitor, for Metastatic Castration-resistant Prostate Cancer and DNA Damage Response Alterations: TALAPRO-1 Safety Analyses
Source: Oncologist. 2022 Sep 19;27(10):e783–95. doi: 10.1093/oncolo/oyac172 (PMC9526483; doi:10.1093/oncolo/oyac172)

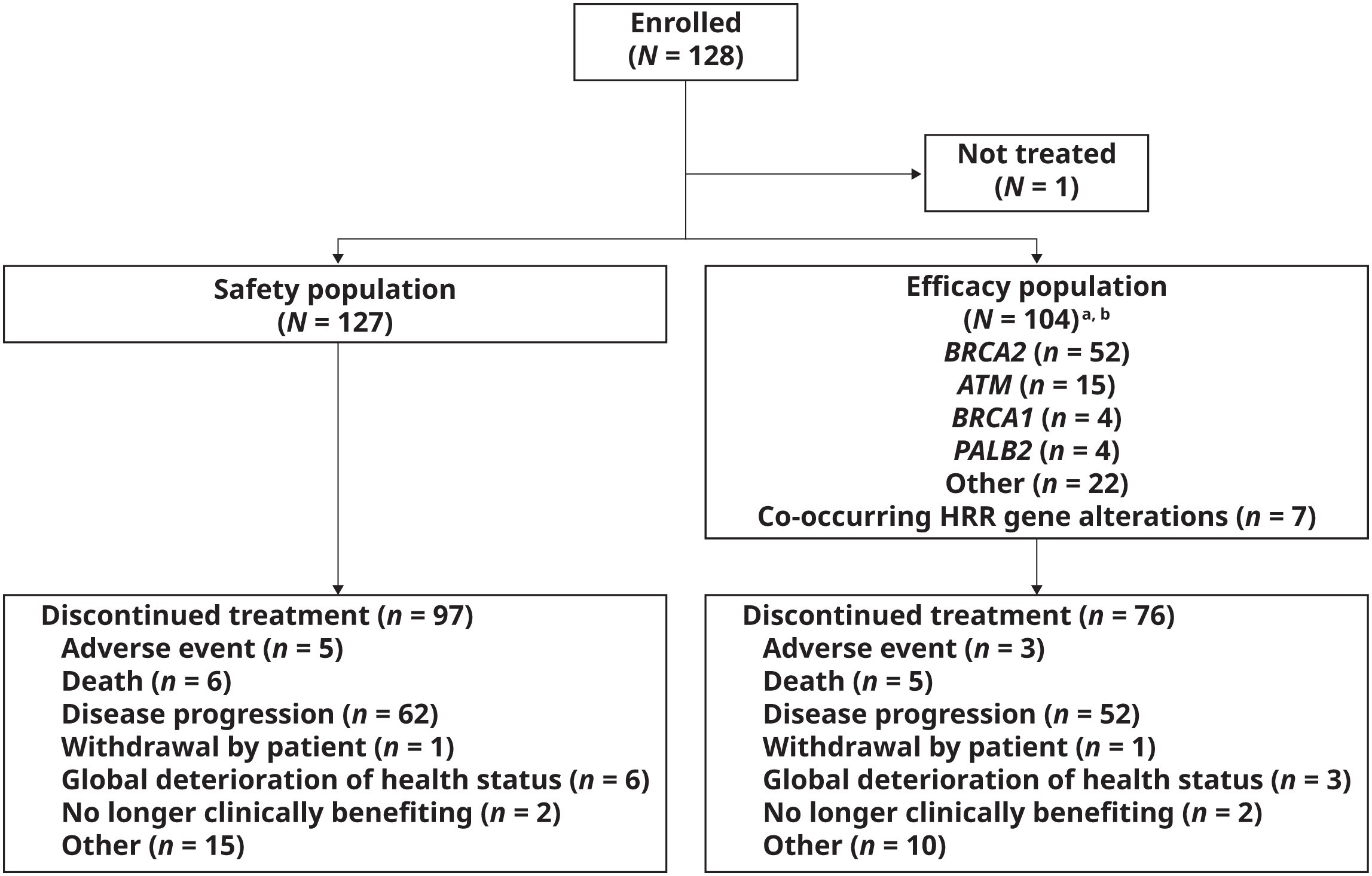

Supplement: oyac172_suppl_Supplementary_Figure_1 [file oyac172_suppl_supplementary_figure_1.jpeg]

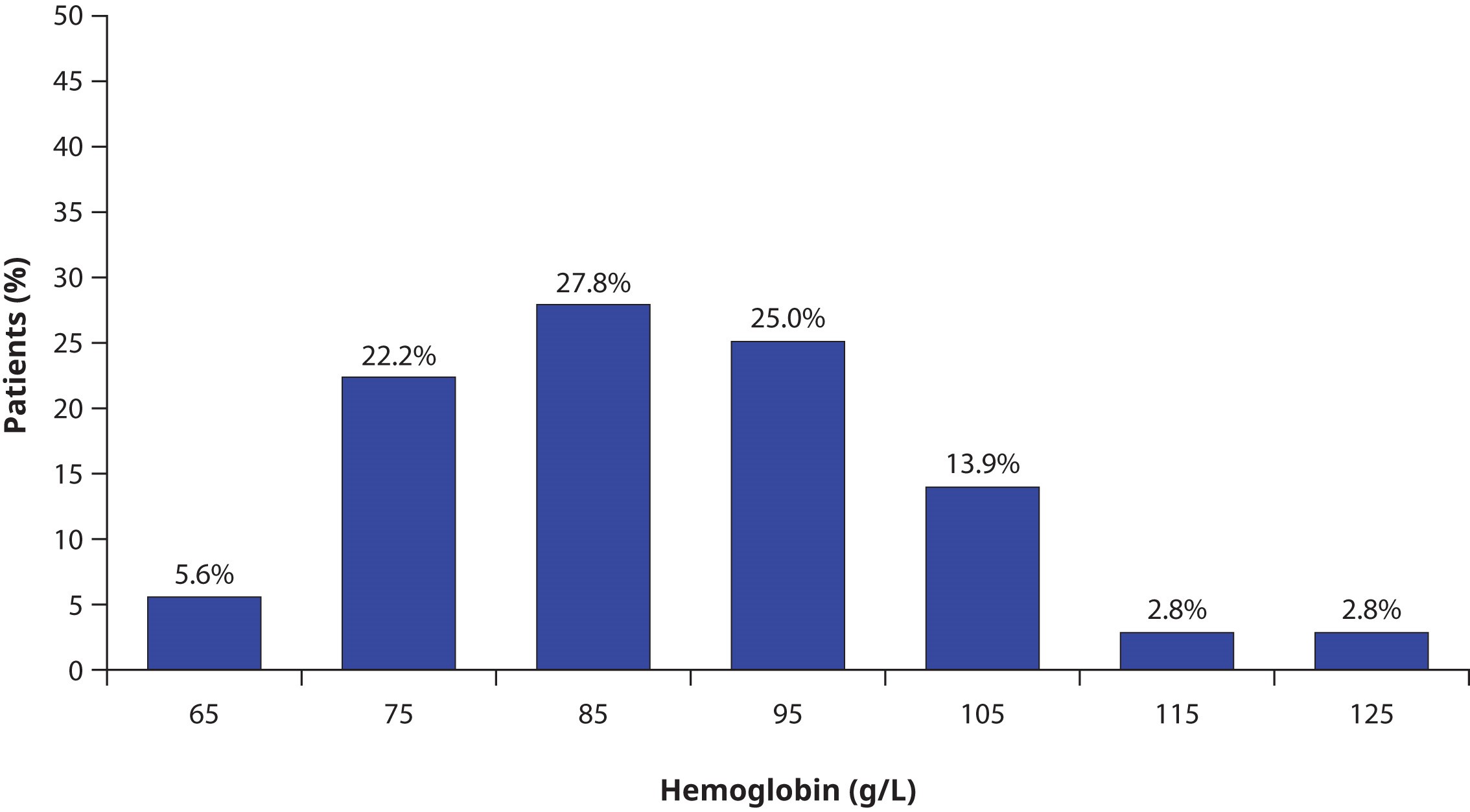

Supplement: oyac172_suppl_Supplementary_Figure_2 [file oyac172_suppl_supplementary_figure_2.jpeg]

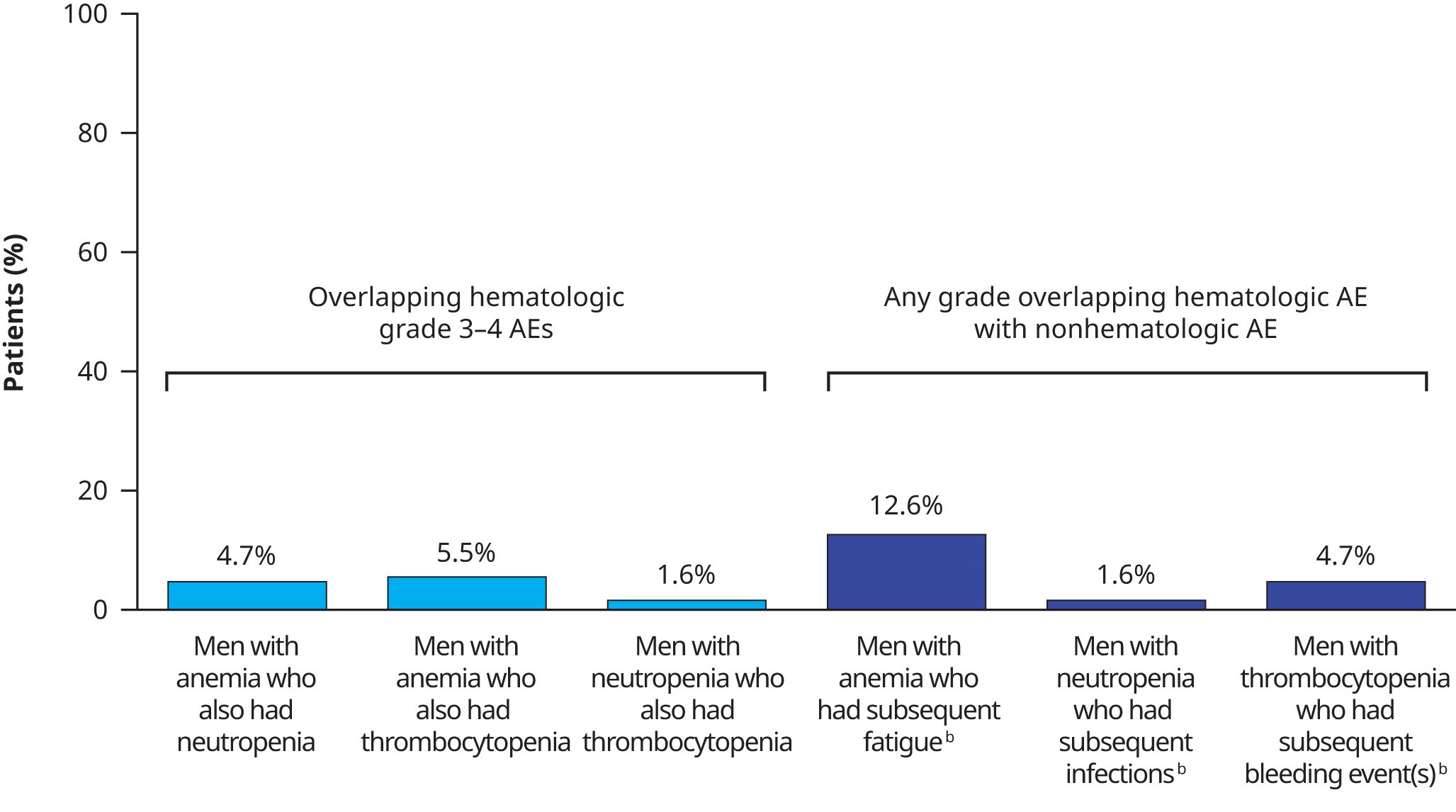

Supplement: oyac172_suppl_Supplementary_Figure_3 [file oyac172_suppl_supplementary_figure_3.jpeg]

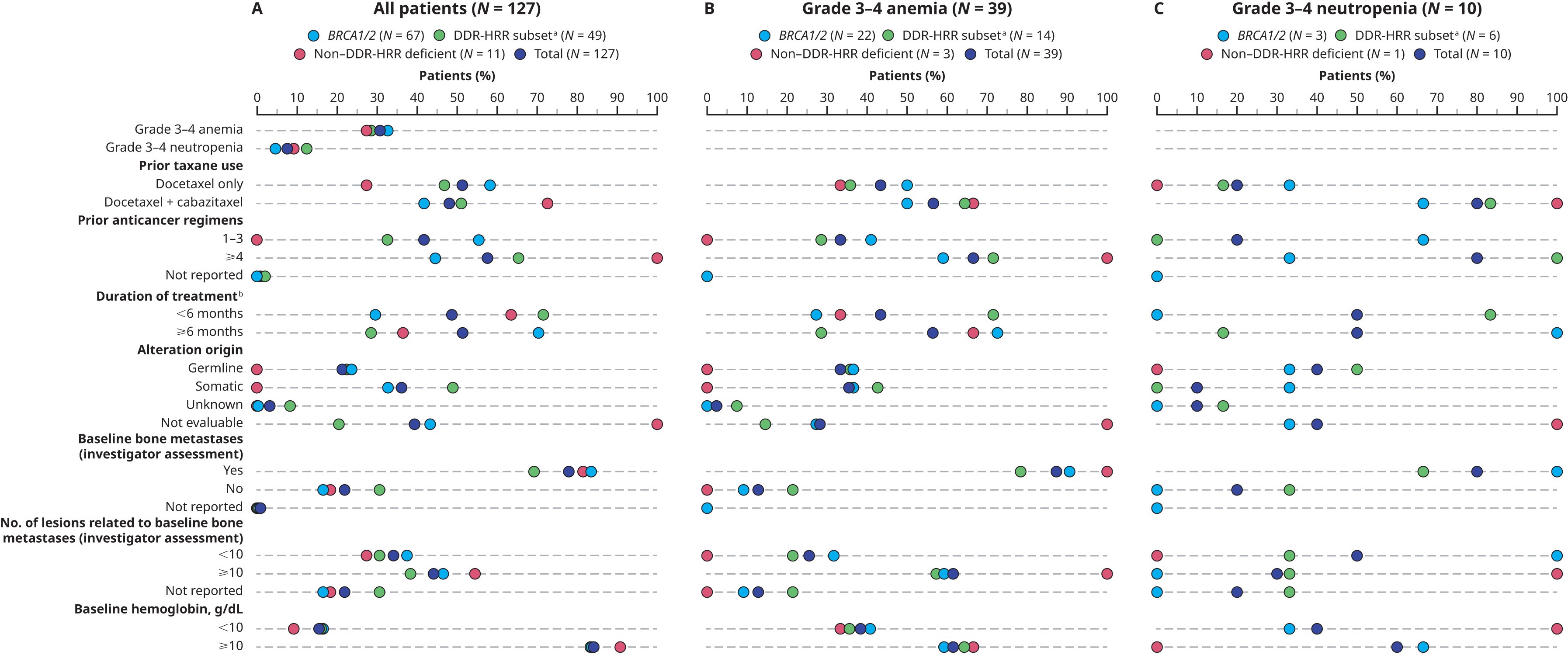

Supplement: oyac172_suppl_Supplementary_Figure_4 [file oyac172_suppl_supplementary_figure_4.jpeg]

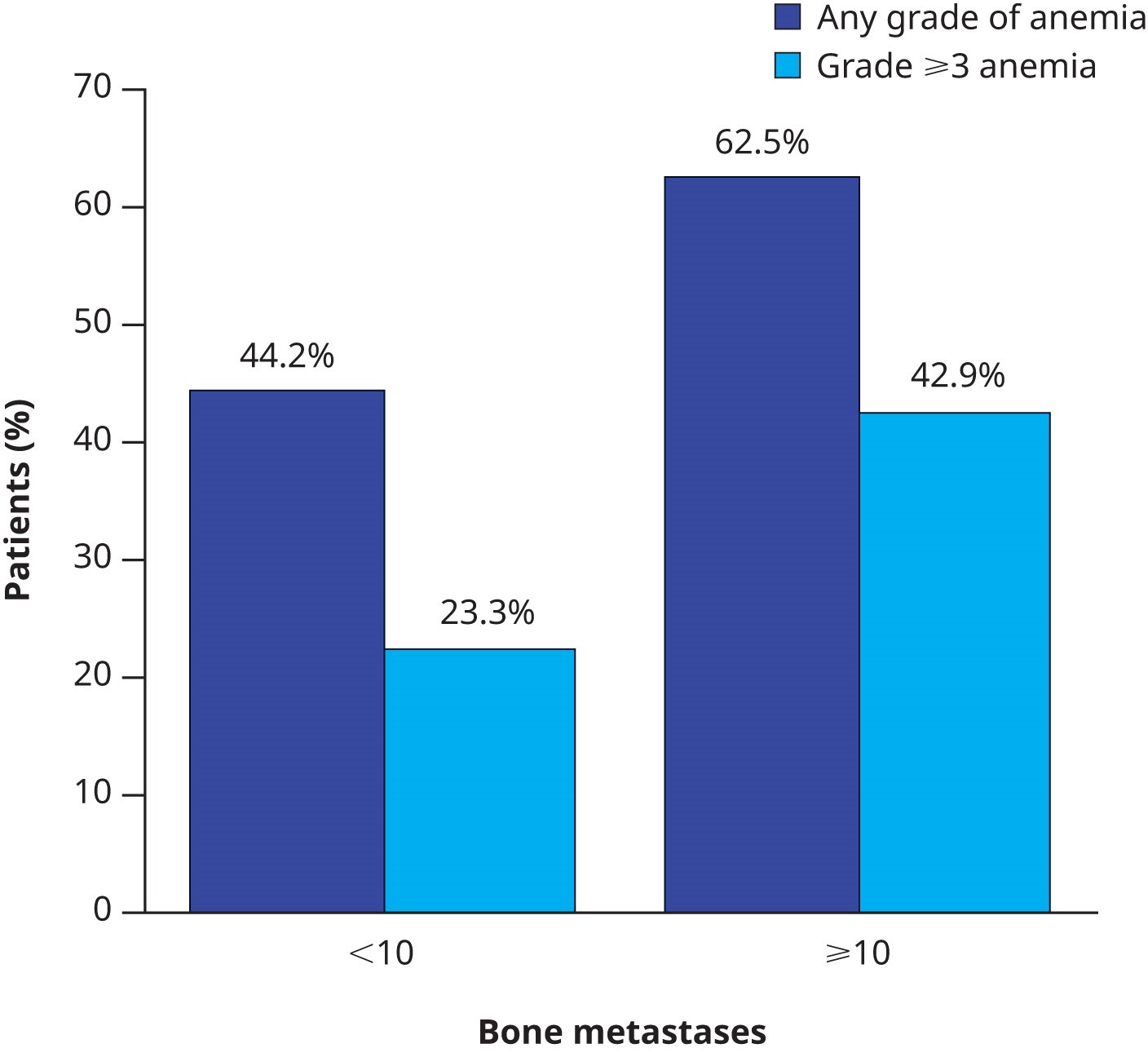

Supplement: oyac172_suppl_Supplementary_Figure_5 [file oyac172_suppl_supplementary_figure_5.jpeg]
