# Supplementary material for: Talazoparib, a Poly(ADP-ribose) Polymerase Inhibitor, for Metastatic Castration-resistant Prostate Cancer and DNA Damage Response Alterations: TALAPRO-1 Safety Analyses
Source: Oncologist. 2022 Sep 19;27(10):e783–95. doi: 10.1093/oncolo/oyac172 (PMC9526483; doi:10.1093/oncolo/oyac172)
Supplement: oyac172_suppl_Supplementary_Figures [file oyac172_suppl_supplementary_figures.pdf]

Supplemental Figure 1. Consort diagram
[a]Efficacy population was the DDR-HRR-deficient measurable disease population defined as all enrolled patients who had measurable soft-tissue disease at screening per investigator assessment, had DDR-HRR alterations likely to sensitize to PARP inhibitor therapy as assessed using a core HRR gene panel (*ATM*, *ATR*, *BRCA1*, *BRCA2*, *CHEK2*, *FANCA*, *MLH1*, *MRE11A*, *NBN*, *PALB2*, *RAD51C*), and had received ≥1 dose of talazoparib.
[b]Gene(s) altered reported using Foundation Medicine results generated either under protocol or by historical results.
Abbreviations: DDR, DNA damage response; HRR, homologous recombination repair; PARP, poly(ADP-ribose) polymerase.

Supplemental Figure 2. Histogram of last hemoglobin level prior to first packed red blood cell transfusion (N = 38[a]; safety population)
[a]Number of patients who received a packed red blood cell transfusion.

Supplemental Figure 3. Most common ADRs with concurrent occurrence (safety population)[a]
AE Grades were evaluated based on NCI-CTCAE (version 4.03).
[a]TEAEs are considered overlapping if a patient experiences both the AEs for at least one day.
[b]For analysis of anemia followed by fatigue, and neutropenia followed by infections, and thrombocytopenia followed by bleeding event(s), the second AE (fatigue, bleeding, infection) had to start the same day or later after the first AE (anemia, neutropenia, thrombocytopenia), but the start date of the second AE was before the end date of the first AE.
Abbreviations: ADR, adverse drug reaction; AE, adverse event; NCI-CTCAE, National Cancer Institute-Common Terminology Criteria for Adverse Events; TEAE, treatment-emergent adverse event.

Supplemental Figure 4. Clinical characteristics of patients in the (A) safety population, (B) with grade 3–4 anemia, and (C) with grade 3–4 neutropenia
[a]Subset includes, but is not limited to, *ATM, ATR, CHEK2, FANCA, MLH1, MRE11A, NBN, PALB2, RAD51C.*
[b]The total number of months from first to and including last day of each study treatment.
Abbreviations: DDR, DNA damage response; HRR, homologous recombination repair.

Supplemental Figure 5. Anemia after treatment with talazoparib in men with mCRPC based on their baseline bone metastases status (N = 127; safety population)
A single patient can have metastases counted for multiple locations.
Abbreviation: mCRPC, metastatic castration-resistant prostate cancer.
