# Supplementary material for: Talazoparib, a Poly(ADP-ribose) Polymerase Inhibitor, for Metastatic Castration-resistant Prostate Cancer and DNA Damage Response Alterations: TALAPRO-1 Safety Analyses
Source: Oncologist. 2022 Sep 19;27(10):e783–95. doi: 10.1093/oncolo/oyac172 (PMC9526483; doi:10.1093/oncolo/oyac172)
Supplement: oyac172_suppl_Supplemental_Tables_1_6_final [file oyac172_suppl_supplemental_tables_1_6_final.pdf]

Supplemental Table 1. Summary of serious TEAEs (safety population)

	**Talazoparib (*N* = 127) n (%)**
Number of patients with serious TEAEs	43 (33.9)
Pulmonary embolism	8 (6.3)
Anemia	5 (3.9)
Disease progression	4 (3.1)
Pneumonia	3 (2.4)
Urinary tract infection	3 (2.4)
General physical health deterioration	2 (1.6)
Pain	2 (1.6)
Platelet count decreased	2 (1.6)
Pyrexia	2 (1.6)
Subdural hematoma	2 (1.6)
Abdominal pain	1 (0.8)
Asthenia	1 (0.8)
Asthma	1 (0.8)
Back pain	1 (0.8)
Bronchitis	1 (0.8)
Bursitis	1 (0.8)
Cancer pain	1 (0.8)
Cardio-respiratory arrest	1 (0.8)
Constipation	1 (0.8)
Dyspnea	1 (0.8)
Dysuria	1 (0.8)
Fall	1 (0.8)
Hematuria	1 (0.8)
Hemianopia	1 (0.8)
Hypotension	1 (0.8)
Malignant neoplasm progression	1 (0.8)
Neoplasm progression	1 (0.8)
Nephrolithiasis	1 (0.8)
Overdose	1 (0.8)
Pain in extremity	1 (0.8)
Pancreatic carcinoma	1 (0.8)
Paresthesia	1 (0.8)
Parotitis	1 (0.8)
Penile pain	1 (0.8)
Pneumonia aspiration	1 (0.8)
Rectal hemorrhage	1 (0.8)
SARS-CoV-2 test positive	1 (0.8)
Sepsis	1 (0.8)
Vomiting	1 (0.8)
White blood cell count decreased	1 (0.8)

Includes data up to 28 days after the last dose of study treatment, or before new systemic (i.e. not including surgery or radiotherapy) antineoplastic therapy, whichever occurred first. Patients were counted only once for specific procedure in the table body. MedDRA v23.0 coding dictionary applied. No men had AML or MDS while on study or by the end of follow-up. Abbreviations: AML, acute myeloid leukemia; MedDRA, Medical Dictionary for Regulatory Activities; MDS, myelodysplastic syndrome; TEAE, treatment-emergent adverse event.

Reprinted from *The Lancet Oncology*, Vol. 22, de Bono JS, Mehra N, Scagliotti GV, et al., Talazoparib monotherapy in metastatic castration-resistant prostate cancer with DNA repair alterations (TALAPRO-1): an open-label, phase 2 trial, Pages 1250-1264., Copyright (2021), with permission from Elsevier.

Supplemental Table 2. Summary of deaths (safety population)

	**Talazoparib (*N* = 127) n (%)**
Deaths[a]	69 (54.3)
Cause of death	
Disease progression	59 (46.5)
Study treatment toxicity	0
Adverse event not related to study treatment	2 (1.6)
Other	3 (2.4)
Unknown	5 (3.9)

[a]No deaths were related to treatment with talazoparib.

Supplemental Table 3. Definitions of hematologic AE grades

	Grade				
**Adverse event**	**1**	**2**	**3**	**4**	**5**
Anemia<br>Hemoglobin (g/L)	<LLN–100	<100–80	<80; transfusion indicated	Life-threatening consequences; urgent intervention indicated	Death
Leukopenia<br>(cells/L)	<LLN–3.0	3.0–2.0	2.0–1.0	<1.0	
Lymphocytopenia<br>(cells x10^9/L)	<LLN–0.8	0.8–0.5	0.5–0.2	<0.2	
Neutropenia<br>(ANC x10^9/L)	<LLN–1.5	1.5–1.0	1.0–0.5	<0.5	
Thrombocytopenia<br>(platelets x10^9/L)	<LLN–75.0	75.0–50.0	50.0–25.0	<25.0	

Based on the Common Terminology Criteria for Adverse Events [35].

Abbreviations: AE, adverse event; ANC, absolute neutrophil count; LLN, lower limit of normal.

Supplemental Table 4. Summary of blood-product transfusions (safety population)

	**Talazoparib (*N* = 127) n (%)**
Any concomitant blood-product transfusion	44 (34.6)
Surgical and medical procedures[a]	44 (34.6)
Packed red blood cell transfusion	38 (29.9)
Platelet transfusion	4 (3.1)
Transfusion[b]	6 (4.7)

Some procedures may have been recorded more than once for an individual patient.

MedDRA v23.0 coding dictionary was applied.

[a]Any concomitant blood product is also listed as a surgical/medical procedure in MedDRA dictionary.

[b]Type of transfusion not specified.

Abbreviations: MedDRA, Medical Dictionary for Regulatory Activities; Reprinted from *The Lancet Oncology*, Vol. 22, de Bono JS, Mehra N, Scagliotti GV, et al., Talazoparib monotherapy in metastatic castration-resistant prostate cancer with DNA repair alterations (TALAPRO-1): an open-label, phase 2 trial, Pages 1250-1264., Copyright (2021), with permission from Elsevier.

Supplemental Table 5. TEAEs leading to dose modifications and discontinuations (safety population)

	**Talazoparib (N = 127) n (%)**
**TEAEs leading to temporary discontinuation of talazoparib**	47 (37.0)
Anemia	24 (18.9)
Platelet count decreased	14 (11.0)
Neutrophil count decreased	10 (7.9)
Decreased appetite	6 (4.7)
Asthenia	2 (1.6)
Pyrexia	2 (1.6)
Arthralgia	1 (0.8)
Back pain	1 (0.8)
Blood bilirubin increased	1 (0.8)
Blood potassium increased	1 (0.8)
Bone pain	1 (0.8)
CD4 lymphocytes decreased	1 (0.8)
Cataract	1 (0.8)
Cough	1 (0.8)
Dehydration	1 (0.8)
Dyspnea	1 (0.8)
Dysuria	1 (0.8)
Ecchymosis	1 (0.8)
Hematuria	1 (0.8)
Hypertension	1 (0.8)
Hyponatremia	1 (0.8)
Liver injury	1 (0.8)
Lymphocyte count decreased	1 (0.8)
Nausea	1 (0.8)
Overdose	1 (0.8)
Pain in extremity	1 (0.8)
Paresthesia	1 (0.8)
Pelvic pain	1 (0.8)
Penile pain	1 (0.8)
Productive cough	1 (0.8)
Pulmonary embolism	1 (0.8)
Rectal hemorrhage	1 (0.8)
Respiratory tract infection	1 (0.8)
SARS-CoV-2 test positive	1 (0.8)
Subdural hematoma	1 (0.8)
Tooth infection	1 (0.8)
Upper respiratory tract infection	1 (0.8)
Urinary tract infection	1 (0.8)
Vomiting	1 (0.8)
White blood cell decreased	1 (0.8)
**Number of patients with TEAEs leading to dose reduction**	33 (26.0)
Anemia	28 (22.0)
Platelet count decreased	6 (4.7)
Neutrophil count decreased	4 (3.1)
White blood cell count decreased	3 (2.4)
Aspartate aminotransferase increased	1 (0.8)
Asthenia	1 (0.8)

Decreased appetite	1 (0.8)
Fatigue	1 (0.8)
**Number of patients with TEAEs leading to permanent discontinuation**	15 (11.8)
Back pain	2 (1.6)
Platelet count decreased	2 (1.6)
Cancer pain	1 (0.8)
Cardio-respiratory arrest	1 (0.8)
Disease progression	1 (0.8)
Malignant neoplasm progression	1 (0.8)
Pancreatic carcinoma	1 (0.8)
Pulmonary embolism	1 (0.8)
Sepsis	1 (0.8)
Spinal cord compression	1 (0.8)
Subdural hematoma	1 (0.8)
Urinary retention	1 (0.8)
Vomiting	1 (0.8)
White blood cell count decreased	1 (0.8)

Includes data up to 28 days after the last dose of study treatment, or before new systemic (i.e. not including surgery or radiotherapy) antineoplastic therapy, whichever occurred first. Patients were only counted once per treatment per event.

Abbreviation: TEAE, treatment-emergent adverse event.

A portion of this table is reprinted from *The Lancet Oncology,* Vol. 22, de Bono JS, Mehra N, Scagliotti GV, et al., Talazoparib monotherapy in metastatic castration-resistant prostate cancer with DNA repair alterations (TALAPRO-1): an open-label, phase 2 trial, Pages 1250-1264., Copyright (2021), with permission from Elsevier.

Supplemental Table 6. Impact of dose reductions on recurrence of TEAEs (safety population)

	BRCA1/2 **(*N* = 67)** ***n* (%)**	**DDR-HRR subset[a] (*N* = 49)** ***n* (%)**	**Non–DDR-HRR Deficient (*N* = 11)** ***n* (%)**	**Total (*N* = 127)** ***n* (%)**
≥1 TE grade 3–4 anemia	22 (32.8)	14 (28.6)	3 (27.3)	39 (30.7)
Recurrence of any TE grade 3–4 anemia	9 (40.9)	6 (42.9)	2 (66.7)	17 (43.6)
**Number of recurrences**				
1	4 (44.4)	2 (33.3)	1 (50.0)	7 (41.2)
2	2 (22.2)	2 (33.3)	0	4 (23.5)
3	2 (22.2)	1 (16.7)	0	3 (17.6)
>3	1 (11.1)	1 (16.7)	1 (50.0)	3 (17.6)
≥1 TE grade 3–4 anemia with normal renal function	12 (17.9)	4 (8.2)	0	16 (12.6)
≥1 TE grade 3–4 anemia with mild renal function	4 (6.0)	7 (14.3)	2 (18.2)	13 (10.2)
≥1 TE grade 3–4 anemia with moderate renal function	6 (9.0)	3 (6.1)	1 (9.1)	10 (7.9)
Baseline hemoglobin ≤100 g/L	12 (17.9)	10 (20.4)	1 (9.1)	23 (18.1)
Incidence of TE grade 3–4 anemia	9 (75.0)	6 (60.0)	1 (100.0)	16 (69.6)
Recurrence of TE grade 3–4 anemia	6 (50.0)	2 (20.0)	1 (100.0)	9 (39.1)
TE grade 3–4 anemia leading to dose reduction	14 (20.9)	8 (16.3)	1 (9.1)	23 (18.1)
Followed by recurrence of TE grade 3–4 anemia	5 (35.7)	4 (50.0)	1 (100.0)	10 (43.5)
TE grade 3–4 anemia leading to transfusion	17 (25.4)	12 (24.5)	3 (27.3)	32 (25.2)
Followed by recurrence of TE grade 3–4 anemia	7 (41.2)	6 (50.0)	2 (66.7)	15 (46.9)

Recurrence was defined as the occurrence of the same adverse event that started after the end date of prior adverse event.

[a]Subset includes *PALB2, ATM, ATR, CHEK2, FANCA, MLH1, MRE11A, NBN, RAD51C.*

Abbreviations: DDR, DNA damage response; HRR, homologous recombination repair; TE, treatment-emergent; TEAE, treatment-emergent adverse event.
